# Full Genome Sequence of a Natural Reassortant H9N2 Avian Influenza Virus Isolated from Domestic Ducks in Jiangsu Province, China

**DOI:** 10.1128/genomeA.00463-13

**Published:** 2013-07-18

**Authors:** Mingjun Zhao, Qingtao Liu, Qing Sun, Wenjun Zhang, Guo Zhao, Min Gu, Xiaoquan Wang, Shunlin Hu, Xiaowen Liu, Xiufan Liu

**Affiliations:** Animal Infectious Disease Laboratory, College of Veterinary Medicine, Yangzhou University, Yangzhou, Jiangsu, Chinaa; Ministry of Education Key Lab for Avian Preventive Medicine, Yangzhou University, Yangzhou, Jiangsu, Chinab; Henan Center for Animal Disease Control and Prevention, Henan, Chinac

## Abstract

In this study, the complete genomic sequence of a novel reassortant H9N2 avian influenza virus (AIV) from domestic ducks in eastern China was reported. Phylogenetic analysis showed that seven of the eight genes were all highly homologous to the chicken-origin H9N2 viruses, whereas the PB2 gene was homologous to the human-origin H1N1 virus, which suggested that domestic ducks might play a key role in the genetic reassortment and evolution of H9N2 AIVs in eastern China.

## GENOME ANNOUNCEMENT

Avian influenza viruses (AIV) are members of the family *Orthomyxoviridae*, which are categorized into 17 hemagglutinin (HA) subtypes and 10 neuraminidase (NA) subtypes, according to the antigenicity of the surface glycoproteins HA and NA (1, 2). Among these, the H9N2 subtype is of great concern, as it has been endemic in poultry populations across Asia and the Middle East and has occasionally been transmitted from poultry to mammalian species (3, 4, 5). Furthermore, phylogenetic analyses revealed that H9N2 viruses were the donors of the “internal” genes of H5N1 viruses in Hong Kong in 1997 (6) and the novel H7N9 viruses in mainland China in 2013 (7, 8). Recent research also demonstrated that H9N2 viruses replicate efficiently in experimental mice without adaptation (9) and transmit via respiratory droplets in ferrets after obtaining the internal genes of 2009 pandemic H1N1 (10). Therefore, the surveillance of H9N2 virus in poultry is needed for us to better understand the ecology and epidemiology of AIV and the potential risk to human health posed by these viruses.

In this study, the H9N2 virus A/duck/Jiangsu/1/2008 (Dk1) was isolated from apparently healthy domestic mallard ducks in the Jiangsu Province of eastern China in January 2008. Using the universal primer set, we determined the complete genomic sequence by reverse transcription PCR (11) and direct sequencing for investigating the detailed genetic characteristics.

The viral genome of this H9N2 virus constitutes eight negative-sense RNA segments, the PB2, PB1, PA, HA, NP, NA, M, and NS genes, with full lengths of 2,341, 2,341, 2,233, 1,742, 1,565, 1,458, 1,027, and 890 nucleotides, respectively. Dk1 carried the amino acid sequence PARSSR/G at the HA cleavage site, a hallmark of low-pathogenic AIV. The receptor-binding pocket of HA1 retained the key amino acid residues Q226 and G228 (H3 numbering), which preferentially bind to the avian influenza virus receptor. However, the PB2 protein had K at position 627, which is characteristic of the mammalian influenza virus and is considered to be critical for the adaptation of avian influenza A viruses to mammals (12, 13, 14), indicating that the virus might have the potential to cross the species barrier to infect humans, so continuous surveillance is required.

The BLAST and phylogenetic analyses of the Dk1 genomic sequence demonstrated that the PB2 gene shared the highest homology (above 99%) with that of a human-origin strain, A/Puerto Rico/8/1934(H1N1), while the other seven genes had the greatest sequence identities (99% to 100%) with a chicken-origin strain, A/Chicken/Shanghai/F/98(H9N2). Therefore, it was speculated that Dk01 may be a novel natural reassortant with its genes from chicken-origin and human-origin influenza viruses, which highlights that domestic ducks as reassortant vessels prompt the genetic reassortment and evolution of AIV in eastern China.

In conclusion, the genome information of Dk1 will contribute to investigations of the segment reassortment mechanism and epidemiological characteristics of H9N2 AIV in eastern China, where the virus is endemic.

### Nucleotide sequence accession numbers.

The complete genome sequences of A/duck/Jiangsu/1/2008 (H9N2) have been deposited in GenBank under accession numbers KF142478 to KF142485.
